# Antifouling Technology Trends in Marine Environmental Protection

**DOI:** 10.1007/s42235-021-0017-z

**Published:** 2021-03-27

**Authors:** Limei Tian, Yue Yin, Wei Bing, E. Jin

**Affiliations:** 1 Key Laboratory of Bionic Engineering, Ministry of Education, Jilin University, Changchun, 130022 China; 2 Weihai Institute for Bionics-Jilin University, Weihai, 264207 China; 3 School of Chemistry and Life Science, Changchun University of Technology, Changchun, 130012 China

**Keywords:** biofouling, antifouling technologies, bionic, non-bionic, biofilm

## Abstract

Marine fouling is a worldwide problem, which is harmful to the global marine ecological environment and economic benefits. The traditional antifouling strategy usually uses toxic antifouling agents, which gradually exposes a serious environmental problem. Therefore, green, long-term, broad-spectrum and eco-friendly antifouling technologies have been the main target of engineers and researchers. In recent years, many eco-friendly antifouling technologies with broad application prospects have been developed based on the low toxicity and non-toxicity antifouling agents and materials. In this review, contemporary eco-friendly antifouling technologies and materials are summarized into bionic antifouling and non-bionic antifouling strategies (2000–2020). Non-bionic antifouling technologies mainly include protein resistant polymers, antifoulant releasing coatings, foul release coatings, conductive antifouling coatings and photodynamic antifouling technology. Bionic antifouling technologies mainly include the simulated shark skin, whale skin, dolphin skin, coral tentacles, lotus leaves and other biology structures. Brief future research directions and challenges are also discussed in the end, and we expect that this review would boost the development of marine antifouling technologies.

## 1 Introduction

Biofouling is a fast, dynamic and complex issue in the shipping industry[1]. Marine biofouling refers to the colonization and settlement of fouling organisms (such as microorganisms, plants, algae, or small animals) on the surface of underwater substrates, which usually seriously affects the normal operation of ships and underwater equipments[2–7]. The shipping industry is the main driver of global trade (close to 90%)[8]. However, due to the attachment of biofouling, it causes billions of dollars in damage to the shipping industry annually[1]. For example, during the voyage from San Francisco to Yokohama, the transportation cost of ships with serious biofouling increased by 77% in 14 days[9]. Due to the increase in the weight and drag of the hull, it is necessary to increase fuel consumption in order to maintain the original sailing speed[10]. According to statistics, the power consumption and fuel consumption of ships seriously affected by fouling have increased up to 86% and 40%, respectively[11–14]. The latter leads to cost increase in the shipping industry, which is estimated to be USD 1 billion per year[8,15]. In addition, the adhesion of fouling organisms make the surface of the hull rough, which not only increase navigation resistance and fuel consumption, but also leads to an increase in harmful gas emissions (including NO$x$, $CO_2$, and SO$x$)[1,16–19]. Smith *et al.* estimate that ship $CO_2$ emissions will increase 50% – 250% from 2012 to 2050[20]. Without further action, the international shipping sector could account for 17% of global $CO_2$ emissions in 2050[21]. Excessive emissions of toxic gases cause the loss of approximately EUR 200 billion annually[22].

According to statistics, there are about 4000 kinds of marine fouling organisms including soft (such as algae and anemones) and hard (such as barnacles, mussels) fouling species (Fig. 1)[6,22,23]. They vary greatly with different geographical locations and different environmental conditions, including salinity, temperature, nutrition, flow velocity, and solar radiation intensity, *etc*[15,24,25]. When the fouling species of a certain sea area are attached to the surface of the ship and brought into the new marine environment, they may grow wildly and disrupt the ecological balance, which will have a crucial

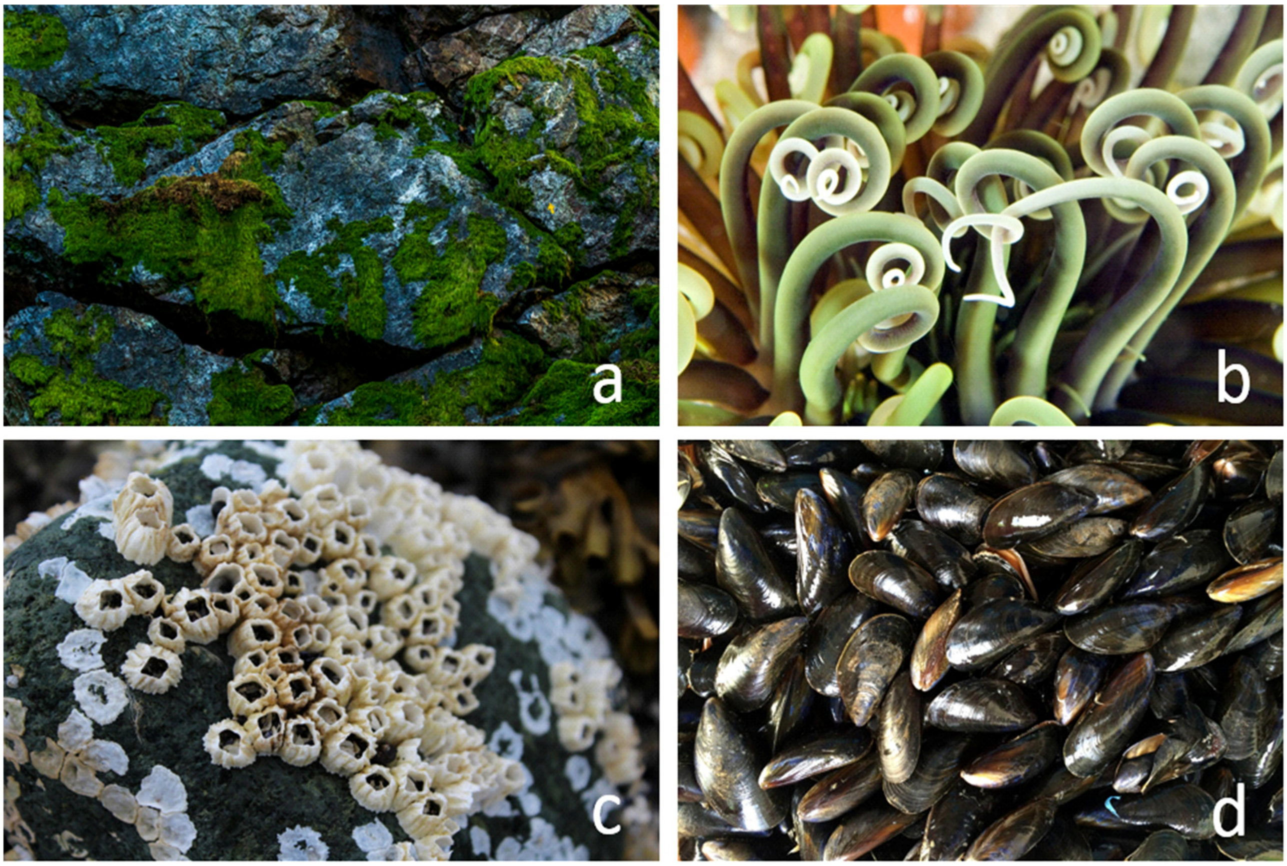

**Fig. 1** (a) Algae (photo by Zdeněk Macháček on Unsplash), (b) Anemones (photo by NOAA on Unsplash), (c) Barnacles (photo by May Gauthier on Unsplash), (d) Mussels (Mussels photo is downloaded from the website of Pixabay (https://unsplash.com/))

impact on the global marine ecosystem[26,27].

Biofouling's growth process can be divided into the following key steps on the timeline, as shown in Fig. 2[28]. First, when the substrate is immersed in seawater, Organic Carbon Residue (OCR) will immediately adsorb on the wet substrate surface and form a conditioned film, which only takes a few minutes. The composition of these OCRs depend largely on the ions, glycoproteins, humic acids and fulvic acids available in the liquid phase[29]. Secondly, a few hours later, under the action of electrostatic force and van der Waals force, bacteria and microorganisms are adsorbed on the conditioned film to form a biofilm[29]. Water flow, Brownian motion, sedimentation and convective motion promote the adhesion of bacteria and microorganisms to the conditioned film[30]. Bacteria and microorganisms attach to the surface through Extracellular Polymeric Substances (EPS), which consist of polysaccharides, proteins, and nucleic acids. Early biofilm growth is an important inducer for the attachment of other complex organisms in the later period. Thirdly, about 7 days later, some protists, single-celled algae spores and marine biological larvae attached to the biofilm surface, which provided nutrition for the formation of large biological fouling communities. Finally, a month or so, substrate surface under seawater has formed a more complex biological fouling communities[31–35].

Antifouling technologies have a long history of development. Humans have been fighting marine fouling for more than 2,000 years[1]. Carve *et al.* first proposed and wrote an article on the impact of biofouling on ship performance[36]. According to literature statistics, the earliest ancient Greek used asphalt, wax, tar and other materials to wrap the hull for antifouling treatment[37]. In the following centuries, plumbum, copper, zinc and other metallic materials were also used as antifouling coatings for hulls[38]. However, the metal material coating is likely to react with seawater, accelerate hull corrosion and shorten the antifouling period. Therefore, in the present day, the metal material

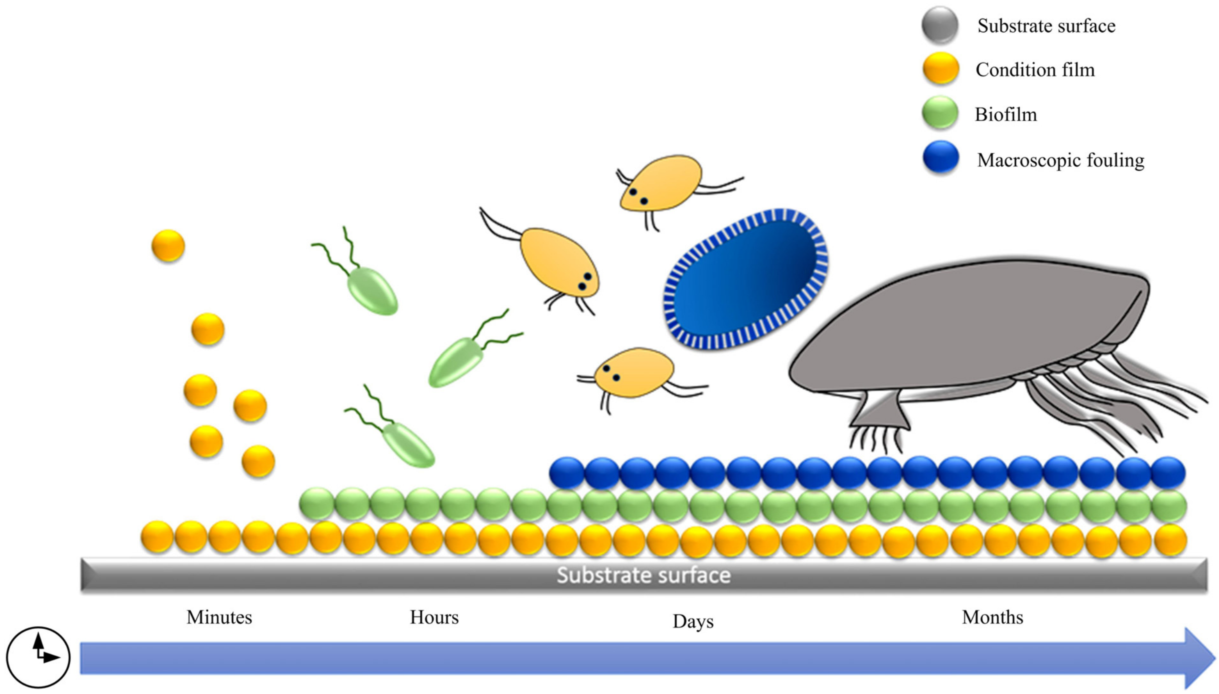

**Fig. 2** Schematic diagram of marine biofouling.

antifouling coatings are not considered the most ideal antifouling method. In recent decades, the researcher looking for new and effective antifouling materials and technologies have became the main direction.

Until the 1960s, the most successful of those antifouling materials were based on alkyl tins, most notably tributyl tin (TBT), which was once considered to be the permanent solution to the problem of marine biofouling[25,39]. However, in subsequent studies, TBT was recognized as an endocrine disruptor to mollusks, causing the growth of male genitalia in female mussels (imposex)[37]. More than that, TBT can also cause shell deformations in oysters, imposex in whelks, and immune response, neurotoxic and genetic effects in other marine species[39,40]. As a result, TBT antifouling coatings were banned worldwide after 2008, following a ban imposed by the International Maritime Organization (IMO) in 2001[5,41,42].

In recent years, laws and regulations on antifouling material compositions are increasingly strict[43]. The European Union issued "Biocidal Products Directive" (BPD) in 2000, which review all biological biocidal products including antifouling paints. Then, it published 46 kinds of antifouling biocides, and there are only 10 kinds of biocides entering the BPD registration procedure. But it has not been determined whether the 10 kinds of biocides can be included in the qualify list. In the meantime, the unqualified biocides will be withdrew permanently from the European Union market. In addition to the European Union, Canada, Sweden and other countries also limited copper release rate on antifouling coatings to protect the marine environment[44].

In order to protect the ecological environment, maintain the ships, marine equipments and facilities normal operation, development of the environmental protection, broad spectrum, and durable antifouling technologies have become a worldwide research focus[45–47]. In this review, we focused on the new principles and new technologies of environmentally friendly antifouling.

## 2 Non-bionic marine antifouling technologies

### 2.1 Protein resistant polymers

As mentioned above, the formation of biofilms and the colonization of fouling organisms are closely related to time. At the beginning of microorganisms adsorption, they strengthen their connection to the substrate surface by secreting metabolites, which are mainly proteins[48]. Therefore, fouling organisms accumulation can be prevented by preventing proteins adhere to the surface of the substrate.

Polyethylene glycol (PEG) is a amphiphilic polymer that has been extensively used in the biomedical and marine environment due to its protein resistance[17,49–52]. The hydrophilic surface of PEG with high surface energies similar to water (72 mN·m$^{-1}$), therefore, the interfacial energy between the surface and water is minimized[53]. This removes any thermodynamic advantage from the adsorption of biomolecules and makes it difficult for biofouling to adhere to the substrate[53–56]. Combined with the anti-protein property of PEG, Ma *et al.* used $SiO_2$ modified PEG to generate a polymer coating[57]. The protein adsorption test was carried out by soaking the polymer coating in the mixed protein suspension. The amount of protein adsorbed on the surface of the polymer coating was lower than the detection standard, which proved that the modified PEG polymer coating had good protein resistance. Similarly, Gan *et al.* also studied the resistance of PEG to protein adhesion. They developed N-isopropylacrylamide of PEG as the copolymer monomer to get nano-particles by free radical deposition method, and the PEG nano-particles can reduce proteins adhere significantly[58]. In the study of protein hydrolysis by PEG, Leng *et al.* found that the free PEG chains can directly interact with protein molecules, which can greatly damage the hydration layer of protein and achieve protein fouling resistance[59]. Kim *et al.* used mussel-inspired polydopamine coating and spin-coating-assisted deposition of PEG catechols[60]. Subsequently, the catechols are crosslinked by $Fe^{3+}$-coordination reactions, thereby fixing PEG to the surface and formed multilayered PEG films that are highly resistant to marine diatom even after immersion in seawater for 4 weeks. Calabrese *et al.* synthesized non-natural amino acids with either an alkyl or PEG side chain that are used to build amphiphilic oligopeptides[61]. When attached to a polystyrene-block-poly(dimethylsilo-xane-co-vinylmethylsiloxane) block copolymer backbone, oligopeptides obviously lower contact angles and improved antifouling performance against *Ulva linza* (*U. linza*). These mixed surfaces generally consist of a polymer backbone with a non-ionic, amphiphilic surfactant attached as a side chain. The non-polar side chains are typically composed of alkyl, fluorinated, or poly(dimethylsiloxane) (PDMS)-based, while PEG is the most prominent polar component studied to date[61].

Although the antifouling performance of PEG is good, it lacks long-term stability and is susceptible to oxidative and microbial degradation[50]. Therefore, recent research has focused on finding alternatives or changing structures, such as zwitterions. The zwitterionic polymer coatings have been extensively used to anti biofouling, it can be attributed to the hydration layer around the zwitterionic moiety[62]. Saffarimiandoa *et al.* developed two polyamide reverse osmosis desalination membranes coated with zwitterionic sulfobetaine silane that compounds including 4-(diethyl(3-(trimethoxysilyl) propyl)ammonia)butane-1-sulfonate (EPBS) and 3- (dimehyl(3-(trimethoxysilyl)propyl)ammonia)propane-1-sulfonate (MPPS)[63]. These investigations using five different marine bacteria isolated from Bosphorus, Turkey to verify it antifouling performance. According to the results, zwitterionic sulfobetaine silane coated membranes showed a strong anti-bacterial effect against the isolated marine biofouling bacteria and significant biofilm adhesion resistance. Venault *et al.* have presented a novel antifouling pseudo-zwitterionic poly(vinylidene fluoride) (PVDF) membrane, using the surface grafting of [2-(Methacryloyloxy) ethyl] trimethylammonium chloride(TMA) and 3-sulfopropyl methacrylate potassium salt (SA)[64]. The PVDF film were synthesize by TMA/SA mixed-charge copolymer *via* plasma-induced surface copolymerization. Through the initial molar content, the surface charge of the film can be well controlled. Mixed-charge covered surface has non-fouling ability and electrostatic repulsion generated by charge bias. Hence, surface-modified membranes can resist protein adhersion and reduce the biofouling caused by the adsorption of *Escherichia coli* (*E. coli*), either exhibit excellent resistance to biofouling in static conditions. Zwitterions offer a promising alternative to PEG, but there have drawbacks which make them difficult to implement. Zwitterion's monomers can be expensive and there are notoriously difficult to work in organic solvents, even interfere or unstable during polymerizations[65]. Bodkhea *et al.* have shown that the amphiphilic and zwitterionic groups on the surface of fouling-release coatings both can improve the antifouling performance of the coatings[66]. Therefore, combined with the existing research results, the antifouling defects of amphiphilic polymers and zwitterionic polymers should be optimized and improved so that they have better antifouling properties and can be widely used.

### 2.2 Antifoulant releasing coatings

Although organotin compounds are banned due to environmental pollution, some antifouling coatings that contained with low toxic antifouling agents are still be used. Copper is the most commonly low toxicity element which used in antifouling agents and usually combined with other organic agents[44,67]. The first idea of using copper in the ships was proposed during the King Christian IV reigned of Denmark in 1618[68], but it only be used in the keel near the rudder[69]. Over the next few centuries, copper has been accompanied with the development of ocean shipping business. Copper is one of the important antifouling materials, but in the early days, people were not sure about its actual antifouling principle[69,70]. Until to the early 19 century, Yebra *et al.* found that cupric ion dissolution in seawater can prevent biofouling attach to substrate when he research the process of copper corrosion[76]. Since then, copper has been formally determined to have antifouling effect[5,71,72]. Antifoulant releasing coating usually used cuprous oxide as the filling material of antifouling agents. The antifouling performance of the coating is greatly affected by the polymer matrix, which refers to the carrier substrate of the antifouling agents. The release types of the polymer matrix determines its antifouling efficiency[7,73,74].

At present, polymer matrix release antifouling agents can be divided into three categories: insoluble matrix coating, soluble matrix coating and self-polishing matrix coating. Insoluble matrix coating, also called contact leaching or continuous contact coating, it cannot decompose or corrode in the seawater[7]. The insoluble matrix mainly contains epoxy resin, acrylate or chloro-rubbers[74]. Insoluble matrix coating contains sufficient soluble antifouling agents. When the antifouling agents particles above the surface coating are dissolved by seawater, and then the secondary antifouling agents particles hidden behind the first layer surface those will keep dissolve and form a continuous antifouling effect[75]. But the solubility of antifouling agents and antifouling agents interparticle contact probability will affect the antifouling effect to some extent. Along with the surface antifouling agents are dissolved, the sub-surface layer antifouling particles need to be indirectly released through the interconnecting gap. If the antifouling agents in the matrix is not uniform or the density are insufficient that will lead to antifouling coating effect decline and reduce the service life. In order to avoid the decrease in antifouling efficiency, which caused by the antifouling agents insufficient solution in seawater, the soluble matrix coating is born. Commonly, development of soluble antifouling matrix coatings need to integrate a large number of rosin (an extract from water-soluble natural resin of pine or fir) or its derivatives. Although rosin is high soluble, the plasticizer mixture is needed to make the final soluble antifouling matrix because of its brittle. Antifouling agents released with soluble matrix and dissolved in water[74]. In dynamic test, the soluble matrix coatings have shown good antifouling performance. But in static test, due to relatively slow dissolution of the coating surface layer and the insufficient release of the antifouling agents, the biological sterilization activity decreased. Therefore, the coating surface is easy to form accumulation of fouling organisms and reducing the antifouling effect[76]. Self-polishing matrix coating generally composed of silicon, copper or zinc ester side groups, it can be easily hydrolyzed in seawater[67]. The matrix's surface can be hydrolyzed by water flow to form new surfaces continuously and release antifouling agents to achieve antifouling effect. Experiment results show that the self-polishing matrix coatings have similar problem to the soluble matrix coatings after a while. When the speed of water flow is insufficient and the coating surfaces update slowly, the coating surfaces will absorb more water and make it thickness accelerated expansion. Therefore, the mechanical properties and antifouling effects of the coatings will be affected[76]. The aging and release rate of antifouling agents under different polymer matrixes are shown in Fig. 3[75,76].

Copper was an effectively and widely used biocide, but only proved to be effective for a period of up to two years[77–79]. Not only that, the widespread use of these (heavy) metal-based antifouling coatings resulted in high-level contamination[79]. In the past decade, the use

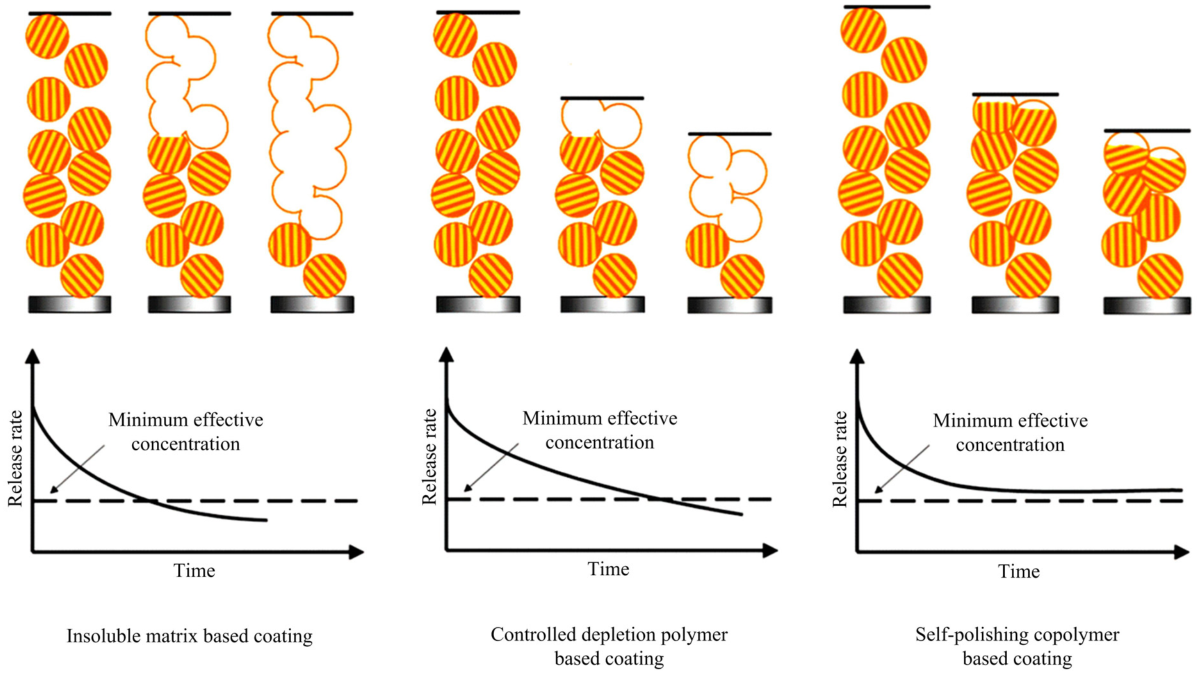

**Fig. 3** The aging and release rate of antifouling agents under different polymer matrixes[76].

of toxic antifouling agents have gradually been restricted, and the search for alternative toxic agents has been advanced. Inspired by these natural systems, researchers have developed new and effective antifouling strategies that are more ecofriendly than conventional strategies, such as the natural compounds. Natural compounds can be extracted from marine microorganisms, bacterias, aquatic plants and invertebrates, which have a small impact on the environment and those are considered to be one of the best alternatives to other traditional toxic biocides[80–85]. Bhattarai *et al*. extracted ethyl acetate from 10 kinds of marine bacteria strains in the east of South Korea sea coast, through spectrophotometer chemical method. Assays showed that chemical compounds extracted from marine bacterias can effectively reduce the density of target strains in the culture medium and the bacteriostatic ability is similar to TBT[86]. In addition to bacteria, aquatic plants are often used to extract natural compounds, such as algae[87,88]. According to statistics, from 1965 to 2012, there were as many as 3,129 kinds of marine natural compounds discovered from algae. Among which about 1,658 kinds of natural compounds extracted from the *Rhodophyta* plants, accounting for 53% of the total and ranking the first. Approximately 1,213 natural compounds extracted from *Ochrophyta* plants, account for 39% of the total in the second place. The *Chlorophyta* extractive only 258 species, accounts for 8% of the total[89,90]. The antifouling compounds of the *Rhodophyta*, *Ochrophyta* and *Chlorophyta* are mostly composed of terpenoids and halogenated compounds. *Asparagopsis armata* is extracted from the algae, that is a kind of small halogenate. The extractive compounds has antibacterial activity to the marine biofouling and biomedical *fungi*, such like *Vibrio*, *E. coli* and *Staphylococcus aureus* (*S. aureus*)[91]. In the study of the *Ochrophyta* (*Sargassum horneri*), Cho *et al*. isolated six similar structure from this compounds using Reversed Phase Liquid Chromatography (RPLC) and High Performance Liquid Chromatography (HPLC) method[92]. Afterwards the structures of these compounds are determined compose of poly pentenyl chain by the Nuclear Magnetic Resonance (NMR) and mass spectrometry analysis. The analytical results reveal those 6 similar compounds have good antifouling activity to combat the typical marine biofouling such as *mussels*, *Ulva pertusa*, *Navicula annexa*.

Although the natural compound antifouling agents showed good antibacterial effect in the test, it is still limited by many factors in practical application. For example, (i) the extraction and synthesis of most natural compounds require large financial support, and large-scale mass production is not yet possible. (ii) Laboratory research data only reflects the antibacterial properties of a single strain. In the face of complex marine environments, the bactericidal properties of natural compounds are still unknown. (iii) Although natural compounds are extracted from marine organisms, in the process of commercialization, they still need to further demonstrate their ingredients and determine unified standards.

### 2.3 Foul release coatings

The adhesion of marine organisms has been historically related to the critical surface tension of the substrate, which was introduced by Zisman[93]. In the literature, an empirical relationship between critical surface tension and the relative amount of bioadhesion has been established, it is commonly known as the Baier curve, as shown in Fig. 4a. The key feature of this curve is that the minimum in the relative adhesion at 20 $mN \cdot m^{-1}$ – 30 $mN \cdot m^{-1}$ does not appear at the lowest critical surface energy. In fact, when the interface energy is at the minimum, the relative adhesion is minimum[35,94,95]. The antifouling coatings with low interface energy are normally called foul release coatings which are different from traditional antifouling coatings, they do not need release toxic agents to achieve antifouling effect, but these coatings minimize the adhesion strength between the fouling organisms and the substrate, so that the organisms can be removed by hydrodynamical stress during navigation or by a simple mechanical cleaning[5,96–98]. Schematic diagram of fouling release from foul release coatings is shown in Fig. 4b.

Under normal circumstances, when the ship coated with a foul release coatings and travels at a speed of 10 knots – 20 knots, fouling organisms will automatically fall off[76,99]. Currently, there are two types of common antifouling coating materials with low interface energy, including polysiloxanes and fluoropolymers, they both exhibit effective functions such as physically hindering the settled biofouling[100]. Fluoropolymers are well-known for their nonpolar nature, which confers a hydrophobic character to their surfaces and a very low critical surface tension or interface energy in the range of 10 $mN \cdot m^{-1}$ – 20 $mN \cdot m^{-1}$[34]. However, fluoropolymers also have many unavoidable defects, such as low structural mobility because of fluorine stiffness that hinders their rotation along the entire polymer backbone and fluorine monomers are relatively expensive, it is difficult to apply fluoropolymers to the hull surface in large areas from an economic point of view[1]. Compared with fluoropolymers, polysiloxanes not only have a lower price, but also have the superior biological, physicochemical, and mechanical properties, which have been utilized in various fields ranging from marine to space applications[1]. With reference to market evaluations, the share of silicone products was USD 17.2 billion in 2017 owing to their extraordinary applications[101–103]. Milne was the first researcher to suggest that polysiloxanes could be used for antifouling materials[104]. polysiloxanes have stable chemical properties and smooth surface. It is not easy to decompose or corrode in seawater, and these features can reduce friction resistance during sailing[105]. However, in the actual application process, the non-polar siloxane chains of the polysiloxane antifouling coating have low bonding strength and poor mechanical properties, it is easy to tear and detach from the substrate under the action of water flow[106]. Otherwise, they are not exhibited significant antifouling effect in static test. For the above problems, Mirabedini *et al.* added $TiO_2$ to adjust the mechanical factors of Silicone Elastic Coating (SEC), such as tensile strength, elastic modulus and wear resistance[107]. Experiments show that the tensile strength, elastic modulus, hardness, abrasion resistance and fatigue strength of the coatings are all increased to about 15% with the increase of $TiO_2$ content. While $TiO_2$ content reached to 10 wt%, its adhesion ability reached a peak but then gradually decreased. This is because of the coatings cohesive strength increased with the increasing of tensile strength and elastic modulus. When the concentration of $TiO_2$ reaches to saturation point, it is difficult disperse for $TiO_2$ due to the polymerization phenomenon in the coating, which leads coating gradually declined the adhesion property for the substrate. To improve the antifouling ability, mechanical properties and self-repairing ability for silicone polymer coatings, Liu *et al.* developed PDMS-based of polyurea groups (PDMS-pUa) which is not only exhibit excellent self-repairing at room temperature or artificial seawater, but also have good adhesion to substrate[99]. The

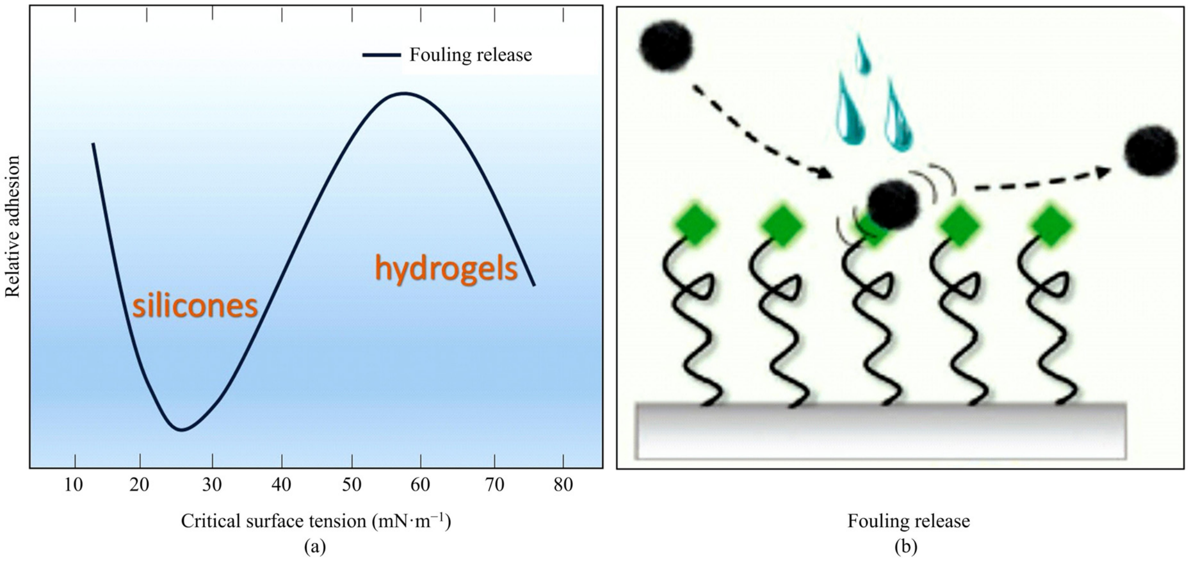

**Fig. 4** (a) Schematic of Baier curve and (b) schematic diagram of fouling release for the foul release coatings[79].

experimental photos for self-repairing samples are shown in Fig. 5. In the experiment, researchers mixed PDMS-pUa with a kind of relatively environment-friendly antifoulants (4,5-dichloro-2-n-octyl-4-isothiazolin-3-one, DCOIT) to form polymeric films, and immersed the polymeric films in the natural seawater for marine field tests. After 6 months, due to its low elastic modulus, low surface energy and continuous release of antifouling agents, the polymer films exhibited extremely low fouling adhesion compared with other samples.

Foul release coatings are regarded as an environmentally friendly antifouling technology. It relies on the physical properties of low surface energy and low elastic modulus to achieve antifouling and it does not dissolve or decompose in seawater. In the current development of antifouling technologies, it plays an important role. However, in view of the existing problems, improving the mechanical properties and antifouling performance under static conditions of the foul release coatings have become the key breakthrough directions for future research.

## 2.4 Conductive antifouling coating

Marine fouling organisms such as bacteria, polysaccharides, and natural organic matter commonly found in seawater, usually have a negative charges, making them susceptible to electrostatic repulsion by a negatively

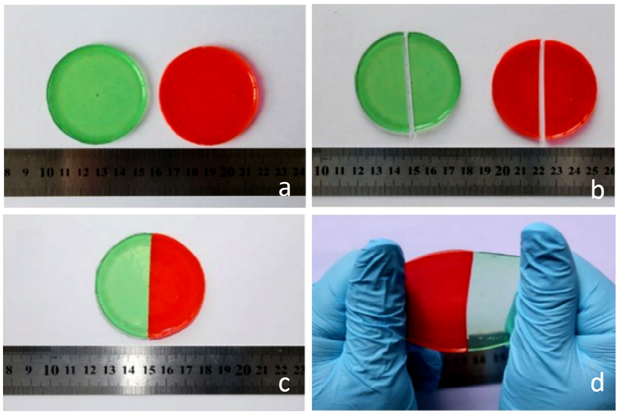

**Fig. 5** Photo of the self-repairing PDMS-pUa films at the temperature of 25 ˚C, and for clear observation, the sample of PDMS-pUa films are dyed in red and green[99]. (a) The original sample; (b) the damaged sample; (c) the self-repairing sample; (d) the self-repairing sample for 48 h.

charged films surface. Conductive antifouling coatings are an electrochemical antifouling method thought increased electrostatic repulsion between the films and the foulants which can reduce the fouling adhesion[108]. Conductive antifouling technology does not produce toxic substances, which is an important environmental protection antifouling technology.

Conductive antifouling coatings usually use ethyl carbamate as the matrix copolymer, and add a mixture of graphite and carbon black as a conductive material. In electricity experiments it was found that black carbon formed a continuous conductive network structure in the matrix copolymers, which can make the coating fully realized the electrical contact between the graphite. In the antifouling experiments, the survival rate of *Vibrio alginolyticus* attached to the conductive coating surface decreased significantly[109,110]. Conductive polyaniline (PAni) is another special conductive antifouling material which formed by PAni and epoxy resin (or polyurethane). These coatings were soaked in seawater for 6 months – 9 months, and less than 10% of the coating surface is attached by fouling organisms. The experiment results show that conductive PAni has the same antifouling effect as the cuprous oxide antifouling agents[111]. Mostafacei *et al*. mixed conductive PAni with epoxy resin and injected nano zinc oxide as additive to synthesize conductive nanocomposite coatings (PAni-ZnO)[112]. According to the studies revealed those conductive polymers suchs as PAni and PAni-ZnO nanocomposite can reduce the settlement of algae and banacles on the substrate. From the assays result, while nano zinc oxide is added to PAni in an amount of up to 2%, its antifouling performance has been significantly improved. In addition, due to the small size of the nanomaterial, it is wrapped in the coating and not easy to be precipitated by seawater, which prolongs the service life of the coating. In the actual marine experiment for up to 9 months, the surface of PAni-ZnO coatings can not form a arge-scale accumulation of fouling organisms. Aldissera *et al*. also used PAni for conductive antifouling studies[113]. They mixed the PAni and HCl to get PAni-ES blending polymer, and put 0.1% mol·$L^{-1}$ $NH_4OH$ solution in PAni-ES polymer to get PAni-EB blending polymer. The conductive polymers with different components are tested by thermogravimetric analysis, salt spray test and marine solid panel test. The results show that conductive polymers have a significant antifouling performance compared to the coating which only containing cuprous oxide. The conductive polymers antifouling performance and efficiency are expected to gradually replace toxic metals coatings such as cuprous oxide. However, while the negatively charged domain formation of a strongly bound rendering the surface antifouling, it also presents some major drawbacks: (i) these negative charges facilitate nonspecific adhesion of positively charged foulants; (ii) due to technical reasons, the current conductive antifouling coating technologies are only suitable for small vessels, and the application for large vessels still need further research.

## 2.5 Photodynamic antifouling technology

In the early stage of the formation of marine biofilm, microbial cells accumulate in the biofilms composed of water, polysaccharides and EPS[114]. Due to multiple factors such as different types of organisms, environmental conditions and nutrient supply, the three-dimensional structure of biofilms varies greatly[115,116]. Commonly antifouling technologies usually use low toxic or nontoxic antifouling agents to kill microorganisms and achieve antifouling effect[116–118]. However, a single antifouling agent cannot have a good antifouling effect on every microorganism in the ocean and it can accumulate in the ecosystem often harm to the marine environment[119]. At the same time, long-term use of antifouling agents may lead to microbial drug resistance, thereby gradually reducing the antifouling performance of the antifouling agents.

Photodynamic antifouling technology combines a non-toxic dye photosensitizer (PS) and harmless, low-intensity light to match the PS absorption peak that generates Reactive Oxygen Species (ROS), leading to intracellular biological molecules (lipids, proteins, and nucleic acids) oxidation. In most cases, microbial cell damage occurs in the cell wall, and the cells are penetrated, thereby selectively killing the microbial cells. Photodynamic antifouling technology can effectively kill different types of microbial cells in the biofilm by changing the solubility, photochemical properties and photophysical properties of the PS[117]. It has broad-spectrum antifouling properties and will not produce drug resistance[117,120]. Through the study of marine biofouling, it was found that most of the plankton bacteria in the ocean are Gram-negative bacteria, and the bacteria in the marine sediments are Gram-positive bacteria. Gram-positive bacteria and Gram-negative bacteria play an important role in the marine microbial environment[121–123]. The results of photodynamic experiments show that phthalocyanine can be used as the PS for Gram-negative bacteria, such as *E. coli* and *Pseudomonas aeruginosa* (*P. aeruginosa*). Minnock *et al.* found that the photosensitivity of Cationic Pyridinium Phthalocyanine (CPP) can inactivate Gram-negative bacteria and the CPP killing the bacterial cells are closely related to the illumination time and dye concentration[124]. Bertolonib *et al.* exposed bacterial cell to a singlet oxidation with PS substrate and verified the bactericidal effects of singlet oxygen on both Gram-positive and Gram-negative bacteria[125]. The results show that the living cell samples are significantly reduced for different time periods. Castro *et al.* added porphyrin to chitosan to form a novel anti-adhesion photodynamic film[126]. The material generates singlet oxygen under visible light irradiation, which can inhibit cell adhesion and biofilm formation. These results provid a foundation for the research of killing microorganisms with different PS under harmless visible light irradiation, and these PS are expected to be applied in antifouling coatings. However, under actual conditions, only a limited amount of visible light can be received under the surface of seawater, which has become a major obstacle to the promotion of this technology.

In addition to visible light, ultraviolet (UV) radiation is also an important choice for photodynamic antifouling. Within the UV spectrum, the UV light wavelength (100 nm – 280 nm) is the most germicidal as it breaks the chemical bonds between DNA and RNA polymers within microorganisms[119,127,128]. Seki *et al.* used *Bacillus subtilis* (*B. subtilis*) spores as biological samples and set up an UV irradiation device in the upstream of the water, then release a quantitative *B. subtilis* spores culture solution though the device. The downstream water was sampled to determine the sterilization rate of the UV irradiation device. The results show that survival rate of *B. subtilis* spores are significantly reduced[129,130]. The application of UV light has been successfully utilized for the prevention of biofouling in seawater systems, instruments and sensors, and has begun to explore the application on ships' hulls[131]. For example, Mackenzie *et al.* used periodic UV-C illumination has been shown to inhibit the accumulation of biofouling on oceanographic sensors[132]. Hunsucker *et al.* investigated the efficacy of using UV light to prevent biofouling in the context of ships' hull coatings. It was found that 1 $\text{min}\cdot\text{day}^{-1}$ UV light exposure was effective at preventing hard fouling development for epoxy coatings. Continuous UV light exposure resulted in no biofouling settlement for copper coatings. Intermittent UV light exposure was effective at preventing biofouling recruitment to both the copper fouling release coatings and the fouling release coatings. In the above studies, although they have good antifouling properties, they still need to be further standardized in terms of power, exposure time, frequency and dosage of photosensitizer for UV treatment. In addition, the transmission of UV light in seawater and the effects of water quality need to be considered, these factors will affect the amount of UV light reaching the surface, thereby affecting the antifouling effect[133].

UV irradiation can not only directly kill microorganisms, but also modify and treat antifouling coatings to assist in the development of antifouling technology. Alves *et al.* activated the surface of the thermoplastic polyurethane (TPU) by UV irradiation. After surface activation, methacrylic acid (MAA) was linked to the surface of TPU in order to improve its reactivity. The results show that the cell adhesion can be reduced by activating MAA through UV irradiation[134]. UV nano-imprint lithography can copy the moth-eye structure onto the cured resin to form the moth-eye microstructure. UV nano-imprint technology has little damage to the mold, and can form a nano-scale high-density polymer structure with antifouling effect[135]. The mutual application of UV technology has been gradually promoted.

## 3 Marine antifouling technology based on biomimetic structure

Bionics (Bionics, Biomimetics, Biomimicry) is defined in many ways, and there was not a fully unified understanding until in 1960, an American military surgeon Jack E. Steele first proposed the concept of bionics[136]. It is a new cutting-edge science and technology that brings together many disciplines such as biology, mathematics and engineering[137]. Bionics is usually defined as a non-toxic, harmless and environmentally friendly method. In recent years, the bionic antifouling technologies have been greatly developed and used in marine industry. Through long-term observation of marine organisms, it is found that even if the plants and animals in the ocean are immersed in water for a long time, the surface is still clean and there is no fouling attachment, such as lotus leaves, aquatic plants, sharks, whales and fleshy corals. Most of these marine organisms can resist the adhesion of biofouling by secreting mucus (including bactericidal substances or reducing drag) or by exhibiting a special structure on the skin's surface[138]. Inspired by the above, people extract antifouling substances from the marine organisms to synthesize natural antifouling agents(for details see in section **2.2**), and mimick the epidermal structure of marine organisms or plants, such as sharks, whales, dolphins, corals, and lotus leaves. Utilizing those proper properties to make biomimetic antifouling coatings, which are beneficial to antifouling[139].

### 3.1 Biomimetic antifouling technology of animal skin structure

#### 3.1.1 Biomimetic shark skin for antifouling application

Large marine animals, such as sharks (as shown in Fig. 6a), whales, dolphins and other non-fouling organisms, their skin surface can secrete a certain viscous liquid. Mucousm found on shark skin and other marine animals are believed to act as a lubricant and further reduce drag with the lower skin friction, hence the shark skin is regard as hydrophilic[140]. Meanwhile, the surface topography for many naturally marine organisms have emerged as the fouling defense mechanism that are seen as hydrophobic[141].

Shark skin is composed of small, curved dentate domains, which are called dermal denticles or placoid scales[142,143], as shown in Fig. 6b. This special skin topography has three-dimensional morphological gradient changes and has better hydrophobicity compared with independent variation gradient structure[144,145]. When sharks swim in the ocean, the zoospore and bacteria are difficult attached to the microstructure surface that is smaller than its own size, resulting in a sharp decline in bacterial adhesion. By imitating shark skin features, Schumacher *et al*. changed the aspect ratio (feature height/feature width) of topographical features in poly (dimethylsiloxane) elastomer (PDMSe) and showing the topography in Figs. 6c and 6d[144]. The spore density of *Ulva* was reduced 42% with each unit increase in aspect ratio of the shark antifouling topography surface. Similarly for *Balanus Amphitrite* (*B. Amphitrite*), the number of barnacle cyprids settled was reduced 45% with each unit increase in aspect ratio. Sakamoto *et al*. performed an antibacterial test using a polypropylene ester plate (width 2 μm, depth 0.4 μm) simulating the shallow groove surface of the shark skin[146]. The results showed that the surface of the shark-like skin morphology has a better anti-bacterial effect on reducing bacterial adhesion. However, numerous studies have shown that real shark skin is much more complex than the simple two-dimensional spinal structure. Carman *et al*. presented the sharklet AF™ surface which is a multi-feature topography consisting of 2 μm in wide rectangular ribs (4 μm, 8 μm, 12 μm, and 16 μm in length) spaced by 2 μm with a height of 3 μm made from a PDMSe[143]. The sharklet AF™ surface can decrease the attachment of *Ulva linza* plankton spores by nearly 86%. Chen *et al.* proposed a fast UV curing method that can transfer and replicate shark skin structure in a large area, and the replication accuracy reach up to 97%[147], as shown in Fig. 6e. After experimental verification, the resistance reduction rate of the coating is improved about 12%, and the antifouling effect is increased by nearly 100 times.

#### 3.1.2 Biomimetic whale skin for antifouling application

In addition to shark skin, pilot whale (*Globicephala melas*) skin also demonstrates very clean surface and free of fouling organism, as shown in Fig. 7a[148,149]. Under observed by freshly freeze-etched skin samples and Bouin-fixed samples which are showing in the Fig. 7b[138]. Baum *et al*. discovered that the surface of pilot whale (*Globicephala melas*) is composed of nanometer-size pores and surrounded by nanoridges, moreover, the surface average pore size is about 0.2 μm$^2$, which are lower than the size of most marine microorganisms[150]. The microorganisms can only attach to the skin surface for a short time, and the attachment of organisms will fall off under water shear[150–152]. Cao *et al.* used a layer-by-layer polyelectrolyte spray method to prepare the coating surface of simulating whale skin structure and showing the surface in Fig. 7c[153]. The surface structure of this coating could be adjusted by changing the pH of poly acrylic acid and polyethylenimine polyelectrolytes. Finally, in the laboratory bioassays showing that surface has good antifouling effect and when the maximum number of nanometer-size pores (about 1 μm) is contained in the smallest structure

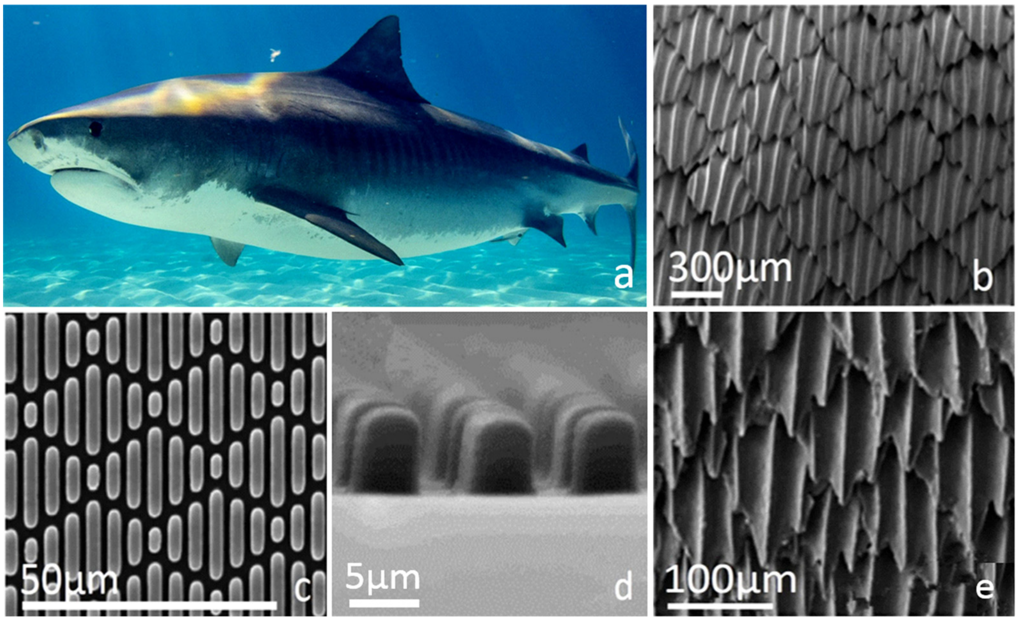

**Fig. 6** (a)The image of the shark (photo by Gerald Schömbs on Unsplash), (b) the Scanning Electron Microscope (SEM) image of a real shark skin surface[142], (c, d) top-down and cross-sectional SEM images of the sharklet AF$^{TM}$ topography produced on the PDMSe[144], (e) a biomimetic reproduction of the shark skin coating under an SEM[147].

(600 nm), the adhesion of microorganisms to the coating surface is the lowest. However, taking technology of freeze-etching resulted in the partition of the skin surface into hydrophilic non-lipidic and hydrophobic lipidic sectors. These sectors varied in thickness and diameter (about 1 μm – 5 μm and 10 μm –100 μm, respectively), indicating the presence of a skin-covering fluid[138]. In subsequent studies, it is found that skin-covering fluid of the pilot whale (*Globicephala melas*) skin contains a variety of hydrolytic enzyme gels[154,155]. Hydrolytic enzyme gels on the pilot whale (*Globicephala melas*) skin surface displays the properties of a viscoelastic solid, which withstands the high shear regimes during swimming and promote the exfoliation of epidermal cells thus realizing the self-polishing process[138]. Baum *et al*. studied on the activity of the enzymes in the pilot whale stratum corneum[155]. They find a kind of a zymogel formed by aggregate-attached enzymes, which is chemically crucial in reducing the attachment of fouling organisms. Combining the physical and chemical properties of pilot whale skin surface is an important break through for bionic antifouling strategy.

### 3.1.3 Biomimetic dolphin skin for antifouling application

Unlike the placoid scales micro structure of the shark skin surface, the surface of the dolphin skin is observed to appear uniformly flat nanostructures under SEM, and the flat and smooth nanostructures cannot stably adhere to the microbial particles. Based on the study of the antifouling mechanism of dolphins, the dolphin's surface has low surface tension and high hydrophobici-

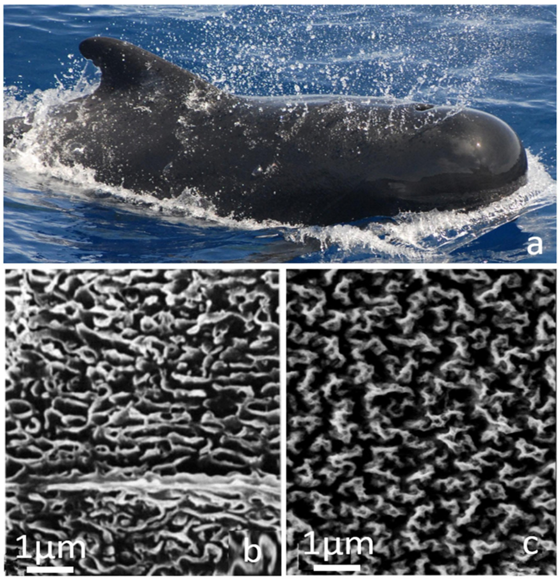

**Fig. 7** (a) The image of the pilot whales (photo by NOAA on Unsplash); (b) pilot whale's nanoridges and enclosing pores structure are demonstrated in the SEM[138], (c) the structure of pilot whale skin surface simulated by polyelectrolyte self-assembly[153].

ty[138,155,156]. Dolphin skin is composed of epidermal layer, dermis and muscle fat layer[157] (Fig. 8a). The special skin structure is considered as a kind of natural flexible wall, which can effectively reduce the disturbance of boundary layer flow and reduce the vibration amplitude under the impact pressure of water flow[158]. The surface of the dolphin skin can secrete more mucus with the increase of water pressure. This hydrophobic mucus helps the dolphins to resist bacterial adhesion with less frictional resistance when swimming at high speed[159].

Although plenty of researches on microtextured coatings fabricated by biomimicking organism surfaces, such as sharks, dolphins have been carried out, there is a practical question worth thinking about that biological skins is susceptive to environment and can't be replicated in a large area. Chen *et al*. choose siloxane modified acrylic resin coatings with positive and negative biomimetic textures replicated from different type of abrasive papers, which combined the contributions of both the micro/nano-structures and low surface energy property[160] (Fig. 8b). They studied the effect of different structure of the coating on the antifouling performance for the common microalgae (*Nitzschia closterium f. minutissima*, *Phaeodactylum tricornutum* and *Chlorella*). The results show that microstructure larger than algae size is easy to accumulated algae, because it leaved microbial life's space to attached, and it does not reflect good antifouling effect, but when the microstructure size is less than the algae size, the surface can effectively inhibit the adhesion of the algae.

### 3.1.4 Antifouling strategies inspired by coral

Most corals are basically kept clean at the continuous erosion of marine biofouling. Therefore, it is assumed that corals can protect against biofouling through secreted chemical composition or by physical antifouling mechanism[161]. Vrolijk *et al*. characterized the natural antifouling surface performance of *gorgonian*[162]. They collected the liquid on the *gorgonian* surface in

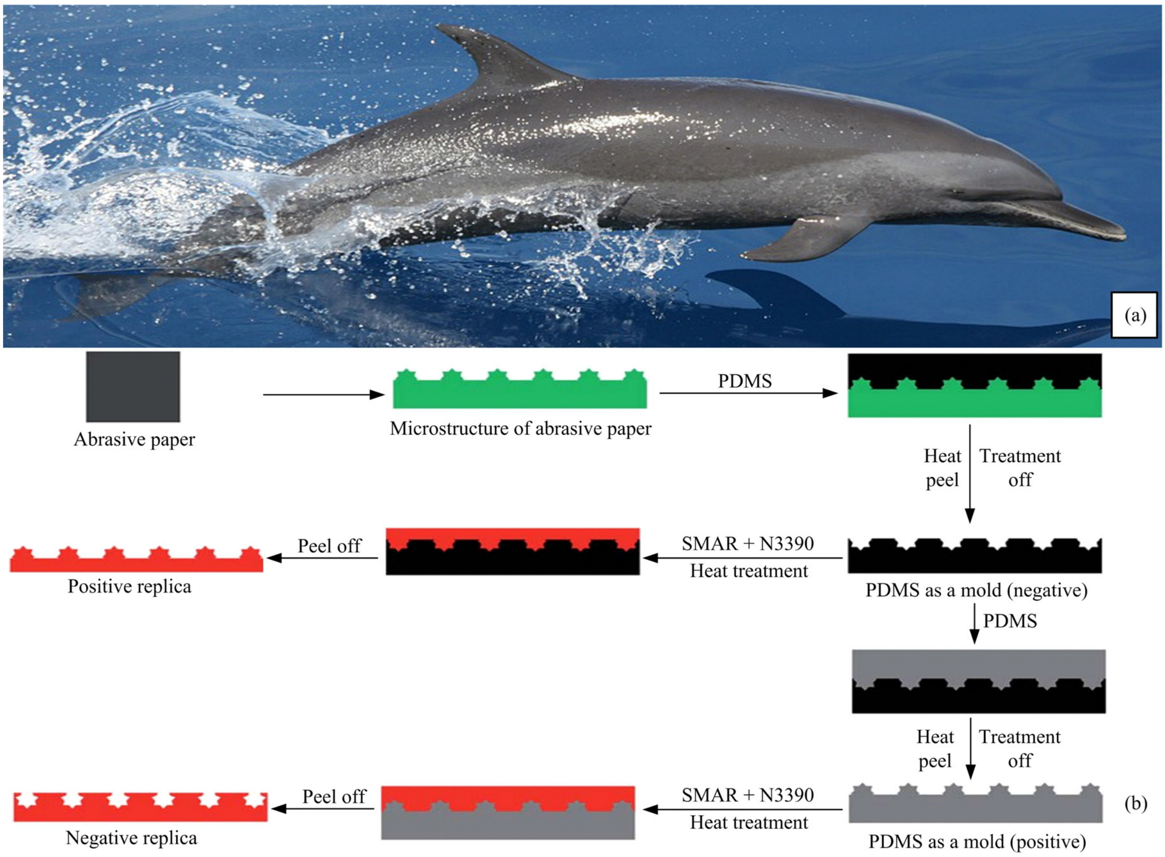

**Fig. 8** (a) The image of the dolphin (dolphin photo is downloaded from the website of Pixabay(https://pixabay.com/)) and (b) the schematic for the creation of textured coatings from the abrasive paper[160].

different seasons, and collected the water around the *gorgonia*, then compared the bacterial activity with two kinds of samples. Finally, they found that the *gorgonia* mucus could inhibit the excessive growth of bacteria. Mucus secreted by different species of corals has different inhibitory effects on bacterial activity and prevention of biofouling. Coral mucus can inhibit the growth of biofilm as well[163]. To verify the coral mucus can have antifouling effect in practical application. Soliman *et al*. extracted seven active material from soft corals, flat corals, cruciferous corals in the eastern seawater of the Alexandria Mediterranean Sea[164]. Then using rosin, chlorinated rubber and other materials as the main matrixes to mix with different kinds of concentrated extracts and prepared antifouling inert coatings. In the bioassays, the coatings were immersed in the seawater for 185 days, and it does not have large-scale biofouling adhere to inert coating, which proved the coral mucus have good antibacterial and antifouling properties. Wang *et al*. extracted seven cembrane diterpenes from soft coral (*Sinularia flexibilis*) and examined the antifouling activity of these compounds to the larvae of the bryozoan (*Bugula neritina*) and the barnacle (*Balanus albicostatus*). The results show that most of the compounds could inhibit biofilms formation and have significant antifouling effects[165]. At present, the most researches on coral antifouling technology is focus on coral secretory. On the one hand, they analyzed the effective antifouling components in coral secretions. On the other hand, they synthesized artificial compounds with similar components by analyzing effective antifouling components in coral secretions. However, there are still a few researches on coral's morphology, mechanical properties and surface structure.

Through observing the surface morphology of soft coral, Tian *et al* found that the skin elastic modulus of soft coral is very low and the skin grow with small tentacles (Fig. 9a)[166,167]. When the tentacles under the low speed ocean that will produce periodic response with fluctuations at a certain amplitude and frequency, which is called "harmonic motion" effect. Following the coral "harmonic motion" principle to achieve antifouling strategy, a coral tentacle-like antifouling film, silicone elastomer composite, was prepared by template method, as exhibited in Fig. 9b. The elastic composite antifouling film generate the effect of "harmonic motion" through fluid flow, which make the surface unstable and provide a basis for inhibiting the adhesion of marine microorganisms, as shown in Figs. 9c and 9d, those manifest the difference methods between non-bactericidal coatings and bactericidal coatings. During dynamic and static bacterial adhesion experiments, the results show that the coral tentacle-like graphene-silicon elastomers antifouling films have been demonstrated strong antibacterial activity against both Gram-negative bacteria (*Paracoccus pantotropha*) and gram-positive bacteria (*B. subtilis*)[166].

In general, imitating the surface topography/structure of marine organisms have demonstrated antifouling performance and achieved excellent results, but there has yet to be a standard topography/structure configuration capable of reducing the settlement of a wide range of marine taxa[36]. Further observation of the topography/structure revealed that the scales of height/depth, width, symmetry, distance between each other and average roughness are important parameters that produce polarization effects for different taxa. As far as the current situation is concerned, it is difficult and impossible to develop a bionic surface topography/structure with the best scale to prevent the settlement of all types of taxa. Nevertheless, exploring the topography and structure have yet to be continue, and may have the potential to prevent fouling from a wider range of marine taxa.

## 3.2 Biomimetic antifouling technology of plant structure

### 3.2.1 Biomimetic lotus leaf surface for antifouling

Lotus leaf have obvious superhydrophobic and self-cleaning property. These characteristics are derived from the roughness of the blade surface. The surface of lotus leaf consists of dense tiny embossed blocks (Fig. 10). The water droplets can roll off easily to remove dust and debris, but not penetrating the microstructure and wetting the surface of the lotus leaf. This surface morphology minimizes interface energy, thus reducing the adhesion between the surface and biofouling, and achieving antifouling effect[162,163,168,169]. Inspired by lotus leaves, Pokroy *et al*. obtained a new roughnessve surface by hot-depositing paraffin wax and fluorinated wax on

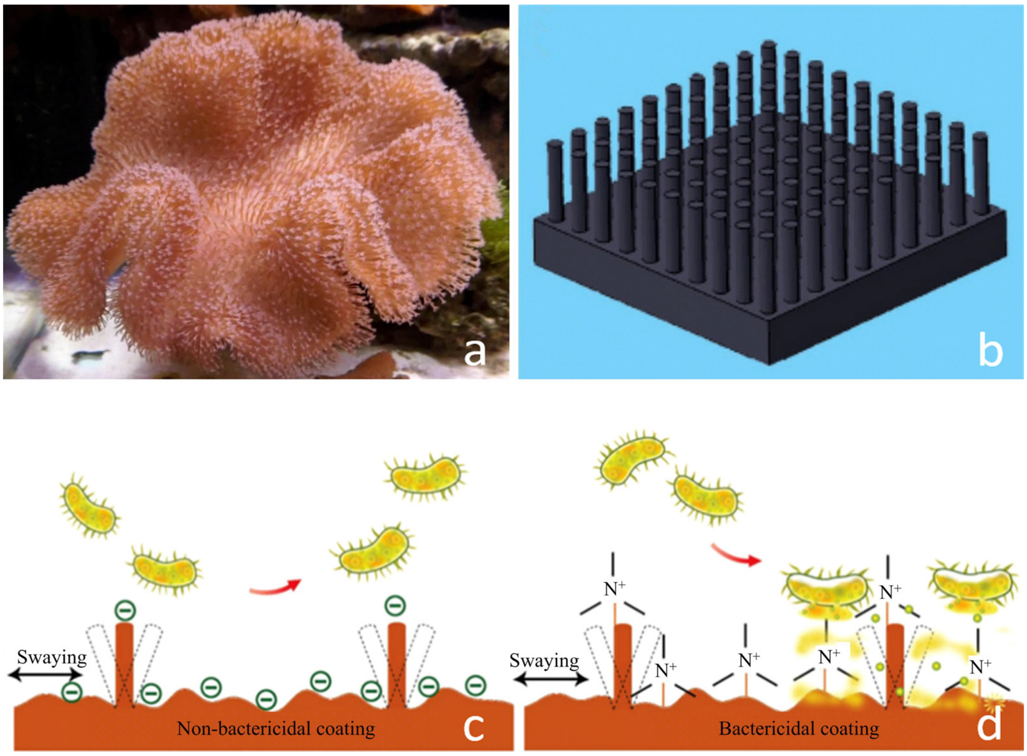

**Fig. 9** (a) The image of soft coral (*Sarcophyton trocheliophorum*), (b) the antifouling coating inspired by soft coral[166], (c, d) illustration of difference between non-bactericidal coatings and bactericidal coatings[166].

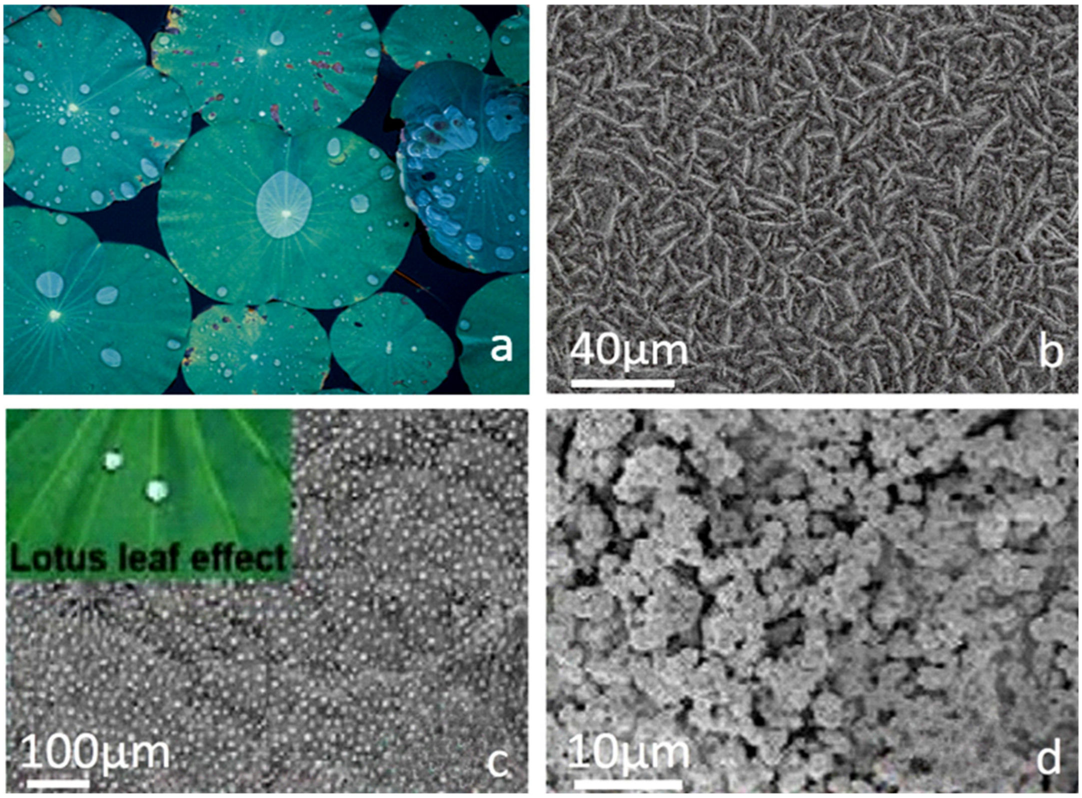

**Fig. 10** (a) The lotus leaf image (photo by Anna Sushok on Unsplash), (b) stratified structure image of hot-deposited wax on copper surface at a magnification of 1000[171], (c) image of particle hierarchy on the surface of real lotus leaf under SEM[173], (d) biomimetic artificial lotus leaf particle hierarchy under SEM[173].

**Table 1** Environmental protection antifouling technologies

| Category | Approach | Main components /The bionic category | Mechanism | Remarks | Ref. |
|---|---|---|---|---|---|
| Non-bionic antifouling technologies | Protein resistant polymers | PEG and zwitterionic polymer coatings | Lower interface energy between the surface and water, resistance to protein adsorption or cell adhesion. | PEG is easy to oxidative and microbial degradation in seawater. Zwitterionic polymers are unstable in polymer matrix and expensive. | [17, 49–66] |
| | Antifoulant releasing coatings | Polymer matrix and antifouling agents | Low toxic or natural antifouling agents released from the polymer matrix. | May harmful to marine environment. Antifouling efficiency decreases during ship stagnation. | [5,7, 67–92] |
| | Foul release coatings | Polysiloxanes, fluoropolymers | Low interface energy, low elastic modulus. | Poor mechanical performance and easy to fall form the hull's bottom. | [1,5,34,35,76, 93–107] |
| | Conductive antifouling coatings | Polymer matrixand conductive materials | Increased electrostatic repulsion between the films and the foulants. | Currently conductive antifouling coatings only can use to the small vessels. | [108–113] |
| | Photodynamic antifouling technology | Photosensitizer, harmless light and UV. | Combining photosensitizers, visible light and UV to destroy microbial cells. | Photodynamic antifouling technology is currently only used in laboratories and has few practical applications. | [114–135] |
| Bionic antifouling technologies | Biomimetic shark skin. | Animal | Shark skin is composed of small, curved, dentate domains, which has better hydrophobicity. | Antifouling films mimicking shark skin structure have well antifouling effect. | [140–147] |
| | Biomimetic whale skin. | Animal | Pilot whale skin is composed of nanometer-size pores, which makes microorganisms hardly attach to the skin surface. | Antifouling films mimicking pilot whale surface have good effect on reducing the microorganism adhesion. | [138, 148–155] |
| | Biomimetic dolphin skin | Animal | Dolphin skin's surface presents a uniform and flat nanostructure, it cannot stably attach microorganism particles. | Antifouling films mimicking smooth dolphin skin have good effect on inhibiting algae adhesion. | [138, 155–160] |
| | Biomimetic coral tentacles | Animal | Coral swinging tentacles make microorganisms hardly attach to its surface. | Antifouling film like coral tentacles shown strong antibacterial activity. | [161–167] |
| | Biomimetic lotus leaf | Plant | Lotus leaf surface composed of dense and tiny embossed blocks. It has obvious superhydrophobic and self-cleaning. | Mimicking the structure of lotus leaf's surface can reduce protein adhesion. | [101,162,163, 168–174] |
| | Flocking anti-fouling technology | Plant | The high-density villi make fouling organisms difficult adhere to the coating surface. | The flocking antifouling coatings have obvious inhibitory effect on invertebrate organisms. | [175,176] |

various surfaces, such as copper, glass and silicon, as shown in Fig. 10b[170,171]. The different waxes formed well-oriented crystal layers with clear nano-morphologies and these new surface morphologies showed superhydrophobic properties. Using natural lotus leaf as template, Zheng *et al*. prepared the lotus leaf polyurethane surface by copying molding method[172]. After the lotus leaf shape is constructed on the surface, the adsorption of protein on the surface is significantly reduced. Chen *et al.* synthesized a flower-like superhydrophobic FOTS-$TiO_2$ particles by solvent thermal synthesis and self-assembly functionalization[173]. The surface morphology of FOTS-$TiO_2$ particles is similar to lotus leaf and showing the photos in Figs. 10c and 10d, which can be used to prepare super hydrophobic coatings on various substrates. From the aspects of waterproofing, mechanical durability, self-cleaning and antifouling performance, the study shows that FOTS-$TiO_2$ coating has strong practicability and versatility, and has more practical application value. Unfortunately, despite this layered superhydrophobic surface development in many research labs, it is often plagued by several limitations, including repellency only toward high surface tension liquids, low mechanical stability, weak pressure stability, low transparency and short-term underwater stability[101]. Breaking the above restrictions will become a major problem in the future.

In addition to lotus leaves, special structures with antifouling effects are also found in rice leaves and butterfly wings. The leaves of rice are long and sinusoidal with parallel grooves, which provide anisotropic flow to the leaves. Besides, the leaves are covered with nano layers formed by the self-assembly of epidermal wax, which provide super hydrophobicity and low adhesion ability. Similarly, the special structure of symmetrical arrangement on the surface of butterfly wings serves the same purpose[174].

#### 3.2.2 Flocking antifouling technology

Flocking antifouling technology is used to implant the smooth fiber nap material into the coating surface through electrostatic action, and the high-density nap is accompanied by seawater vibration. The effect is similar to the thorn of rose, which makes biofouling difficult to be adhere to the coating surface and to achieve antifouling effect[175].

Flocking antifouling technology was first proposed by Swedish engineer Kjell and applied for a U.S. patent[176]. Phillippi *et al.* placed flocking plastic sheets of polyvinyl chloride (PVC) at the U.S. Westport river for one month[175]. The experiments show that flocking plastic sheets have significant inhibitory effects on invertebrates. However, in practical application, due to the complexity of flocking technology, it is difficult to carry out the repair work in the later stage and there is still no standard data conclusion on the relevant parameters of flocking technology, such as villus length and villus density. There are still many obstacles in the application of real sea.

## 4 Conclusion

This paper reviews the research progress of environmentally friendly antifouling technology in the contemporary era (2000–2020), mainly divide the contemporary antifouling technologies into non-bionic antifouling technologies and bionic antifouling technologies.

Non-bionic antifouling technologies mainly includes protein resistant polymers, antifoulant releasing coatings, foul release coatings, conductive antifouling coating and photodynamic antifouling technology. Among them, protein resistant polymers are a preventive environmental antifouling technology, because those can hinder the adhesion of proteins and inhibit the development of biofouling in the substrate. But in the actual experiment, the coating in the water will be accumulated by sea mud and form a layer of thin film after a period of time, and the coating surface resistance is covered by marine mud, then antifouling ability lost gradually. The antifoulant releasing coatings release the low toxic cuprous oxide agent or non-toxic natural antifouling agent through the different polymer matrixes, and gradually spread it in the sea water to achieve antifouling effect. However, with the passage of time, the polymer matrix will face the problem of low antifouling efficiency due to the end of antifouling agents release and insufficient dissolution of the coatings. Not only that, low toxic antifouling agents still pose a threat to the marine environment. Natural antifouling agents are seen to the most ideal by extracting effective antifouling components of marine microorganisms and aquatic plants. However, the effective activity of natural antifouling agents have higher requirements for marine environment, and it still have no broad-spectrum applicability according to the current research status. Different from traditional antifoulant releasing coatings, foul release coating is no longer through release of antifouling agent for antifouling, and the characteristics of low interface energy and low elastic modulus make microorganisms difficult to stay on the surface and hinder the formation of biological fouling. Foul release coatings of fluoropolymer are less used due to their high cost. Polysiloxanes have the same low interface energy and low elastic modulus characteristics, which make them become the mainly foul release coating matrixes at present. However, the polysiloxanes have a weak adhesion to the bottom of the hull, and they are easy to fall off under the scouring water flow. Besides that, polysiloxanes mechanical properties are poor too, they will be easy to tear under the water, so these weaknesses need to be improved in the further. Up to now, there are few reports about the practical application of conductive antifouling technology and photodynamic antifouling technology. But according to many experiments, these two methods have excellent antifouling effect, and belong to the new antifouling technology of non-toxic, environmental protection and economic. However, there are still many problems in practical application. For example, conductive antifouling technologies are only limited used to small vessels and the scope of application of the photodynamic antifouling technology have limitation too. Therefore, they have a great practical significance to continue explore and improve the existing antifouling technologies.

Bionic antifouling technologies mainly include mimicking animal and plant's skin structure to produce antifouling effect. Experimental results show that except for extracting effective natural antifouling components, mimicking animals and plant's skin structure is also an important part of bionic antifouling. This paper reviews the four important marine animal of shark, whale, dolphin and coral. The structure of the shark skin is analyzed under an SEM, it is found that the shark's skin surface has a placoid scales structure, which has better hydrophobicity. The whale surface presents a micro nano-pores structure and it is covered by its own secreted mucus, which blocks the attachment of fouling organisms. Dolphin skin surface is smooth and soft, and it can reduce the frictional resistance and inhibiting the adhesion of biofouling. Coral tentacle can produce a "harmonic motion" effect with wave fluctuations, effectively reducing the adhesion of biofouling. Various antifouling coatings are prepared by mimicking the skin surface structure of four kinds of marine animals, all of those exhibited good antifouling properties in the experiments. In the biomimetic antifouling technology of plant structure, taking the lotus leaf as an example, its surface is composed of dense tiny embossed blocks, which has super-hydrophobic and self-cleaning features. But in the face of complex biological communities in the ocean, a single biomimetic structure is difficult to fight. Increasing the complexity of the biomimetic structure, making it suitable for a wider antifouling space, has become an important choice for bionic antifouling.

In summary, contemporary environmentally friendly antifouling technologies all exhibit good antifouling performance, but are also accompanied by many limitations. With the continuous development of the global economy, the marine industry will also expand year by year. In future research, environmentally friendly antifouling technology needs to be optimized by combining existing technologies and materials to form a broader spectrum and more valuable antifouling technology, which will be important for future marine antifouling the role.

## Acknowledgment

The authors are grateful for grants received from the National Natural Science Foundation of China (Grant No. 51875240), the Jilin Provincial Science and Technology Development Plan, Young and Middle-Tech Leading Talent and Team Project (Grant No. 20200301013RQ), the Department of Science and Technology of Jilin Province (Grant No. 20190103114JH), Key Laboratory Fund of National Defense Science and Technology (Grant No. 6142005190201).

**Open Access** This article is licensed under a Creative Commons Attribution 4.0 International License, which permits use, sharing, adaptation, distribution and reproduction in any medium or format, as long as you give appropriate credit to the original author(s) and the source, provide a link to the Creative Commons licence, and indicate if changes were made.

The images or other third party material in this article are included in the article's Creative Commons licence, unless indicated otherwise in a credit line to the material. If material is not included in the article's Creative Commons licence and your intended use is not permitted by statutory regulation or exceeds the permitted use, you will need to obtain permission directly from the copyright holder.

To view a copy of this licence, visit http://creativecommons.org/licenses/by/4.0/.
